# Aerobic Treatment Units for Widespread Onsite Wastewater Treatment in Coastal Louisiana: Pollution, Policy, and Market-Based Solutions

**DOI:** 10.1021/acs.est.5c17284

**Published:** 2026-05-14

**Authors:** Anmol Soni, Banna Das, Matthew Brand, Aaron Bivins

**Affiliations:** † Department of Public Administration, 5779 Louisiana State University, Baton Rouge, Louisiana 70803, United States; ‡ Department of Civil & Environmental Engineering, 5779 Louisiana State University, Baton Rouge, Louisiana 70803, United States

**Keywords:** ATU, water quality, Lake Pontchartrain, nutrients, governance

## Abstract

Onsite wastewater treatment systems are known to be sources of aquatic pollution; however, scarce data usually prevent system-level assessments. Aerobic treatment units (ATUs) are widely utilized in Louisiana, where groundwater and soil conditions limit septic tanks. By combining an ATU permitting record with housing unit build data, we estimate there were 412,552 permitted ATUs in Louisiana by the end of 2023. We conservatively estimate the annual surface water loading from ATUs in the 24 coastal parishes is 7.5 million pounds of nitrogen and 2.2 million pounds of phosphorus, which are equivalent to 48% and 84%, respectively, of all the nitrogen and phosphorus discharged by major wastewater treatment plants in Louisiana. Despite the use of ATUs for 73.7% of new homes built from 1990–2016, our analysis of policy documents indicates that ATU management in the two coastal parishes with the highest number of ATUs is best described as “basic” with limited compliance monitoring, enforcement, and public awareness. Simultaneously, we estimate that the deployment of Environmental Impact Bonds premised on nutrient recovery and optimized energy consumption could be sufficient to fund routine ATU inspection and maintenance programs. Our findings strongly suggest that the onsite wastewater treatment status quo jeopardizes water quality at scale, demanding the pursuit of engineering and policy solutions.

## Graphical abstract

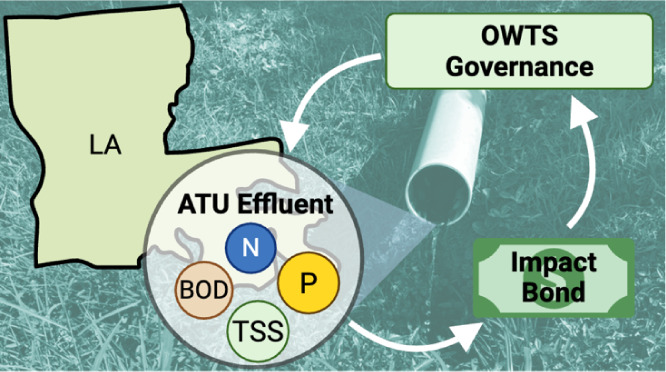

## Introduction

The last national-level effort to quantify the use of onsite wastewater treatment systems (OWTS) in the United States (U.S.) was completed in 1990 and indicated that 1 in 5 households were served by an OWTS.^1^ Data from the more recent American Housing Survey (AHS) suggest 1 in 6 households rely on OWTS, but the AHS is not national in scope and underrepresents OWTS prevalence in rural areas.^2^ The most recent study, using results from pilot testing of a proposed sewer question for the American Community Survey, estimates 32.2 million U.S. households (1 in 4) use an OWTS for their domestic sewage treatment, implying that the number estimated by AHS underestimates by at least 12 million households.^3^ In some states, the percentage of homes served by an OWTS exceeds 40%, including New Hampshire, Maine, Vermont, North Carolina, South Carolina, Kentucky, West Virginia, Alabama, and Mississippi.^4^ In at least seven states, the OWTS usage rate for new housing construction has recently exceeded 50%.^5^ The exact number of systems in use and their spatial distribution, like most details concerning OWTS, are largely unknown due to their fragmented and disjointed governance, which spans hundreds of state and local agencies.^6,7^ Despite the uncertainties, it is likely that OWTS in the U.S. discharge upward of 9.6 billion gallons of effluent each day, enough to fill roughly 10 Olympic swimming pools every minute.

Daily OWTS discharges far exceed the permitted discharges of even the largest publicly owned treatment works in the United States, yet no federal agency regulates OWTS. Instead, these systems are governed at the state and local levels, with some federal guidance. For example, the U.S. Environmental Protection Agency (EPA) has published an *Onsite Wastewater Treatment Systems Manual*,^4^ *Voluntary National Guidelines for Management of Onsite and Clustered (Decentralized) Wastewater Treatment Systems*,^8^ *Handbook for Managing Onsite and Clustered (Decentralized) Wastewater Treatment Systems*,^9^ and *Decentralized Wastewater Treatment Systems: A Program Strategy*.^10^ From state to state, there are large variations in governance structures and responsibility, with health departments assuming primary responsibility in 41% of states, counties in 27%, and the state itself in 19%.^5^ Exact permitting requirements for OWTS within each state vary according to use (residential or commercial), flow rate (gallons per day), and discharge location (surface or subsurface).^5^ In general, OWTS governance is characterized by significant resource constraints, with little to no enforcement beyond the initial installation permitting and inspection.^11^ These challenges can be particularly acute in unincorporated areas where OWTS are more prevalent and county government revenue is limited.^12^ Most often, federal and state programs, such as the SepticSmart Program, emphasize homeowner education to promote proper care and maintenance of onsite systems.^13^

Even with programs such as SepticSmart, many homeowners remain unaware or uninformed about OWTS maintenance and the impact of OWTS failure on local water quality.^14,15^ Deferred maintenance and treatment failure are common among OWTS.^16^ The U.S. EPA estimates septic system lifetime failure rates of 10 to 20%;^17^ however, septic system failure rates in North Carolina have been estimated to be as high as 10 to 20% per year.^18^ For more sophisticated mechanical systems, such as the aerobic treatment units (ATUs) being widely used in Louisiana,^19^ inspection programs have documented an average failure rate of 57.6% across multiple watersheds (Nicole Cormier, personal communication). OWTS failure rates in coastal regions are likely to increase due to increased flooding and sea-level rise, driven by a warming climate.^20,21^ Definitions of “failure” can vary by setting and study, making aggregation and comparison of data across locations challenging. But no matter the definition of failure, if dysfunctional OWTS proliferate, they can become a significant source of anthropogenic pollution, jeopardizing both human and ecosystem health.^7^ Failing septic systems have been linked to sewage contamination in private wells,^22,23^ diarrheal disease among children,^24^ and nutrient loading into coastal waterways.^25^

On a case-by-case basis, there are well-established links between failing OWTS and many negative outcomes, but innovation in both treatment technologies and management paradigms is constrained by available funding.^26^ The primary vehicle for Federal investment in wastewater infrastructure is State Revolving Funds (SRF), which, in some states and certain cases, may allow low-interest or no-interest loans for OWTS maintenance or replacement. Through the Closing America’s Wastewater Access Gap Initiative, the U.S. EPA provides technical assistance to communities with failing OWTS at no cost, and the U.S. Department of Agriculture (USDA) also makes low-interest or no-interest loans and grants available for OWTS repair or replacement for low-income households through several programs. However, the administrative requirements of these programs frequently inhibit participation from households and communities in low-resource and disadvantaged settings where failing OWTS are often found.^27^

The end result of these many barriers is the widely accepted status quo in onsite wastewater treatment – low wastewater treatment service levels, low willingness to pay, low investment, decentralization, and no definitive scope or solution.^28–30^ Pioneering new solutions in the space requires reframing both policy and financial aspects of onsite wastewater treatment to reflect the many environmental services and economic benefits delivered by functional engineered systems. However, risk assessments and benefit-cost analyses of OWTS at scale have been impeded by the availability of data, especially at scale.^6^ Traditional disciplinary silos have also slowed the deployment of sociotechnical analyses to drive innovation in onsite wastewater treatment paradigms.^31–33^

## Materials and Methods

Here, we describe a multidisciplinary approach to (1) estimate the number of ATUs in use in coastal Louisiana, (2) quantify pollutant loadings likely attributable to them, (3) characterize the enabling policy environment, and (4) consider innovative market-based solutions. Our goal is to conduct a systems-level analysis to identify points of failure in the OWTS sociotechnical system, quantify the water quality consequences of such failures, and identify opportunities to leverage economic tools to drive resources toward failure points.

### Study Setting

With 17.5% of its surface area covered by water, Louisiana serves as a critical interface between terrestrial and aquatic ecosystems.^34^ The state covers over 65,000 miles of rivers and streams, 5.5 million acres of wetlands, and 4.9 million acres of estuary,^35^ including Lake Pontchartrain, the second largest estuary in the United States.^36^ Louisiana is also home to the seventh-largest river delta in the world, the Mississippi River Deltaic Plain,^37^ where the Mississippi River discharges water and its contents originating from its more than 815 million-acre drainage basin, roughly two-thirds of the United States.^38^ Louisiana’s aquatic habitat is one of the most important coastal ecosystems in the world, valued for its ecological, cultural, recreational, and economic significance. However, the functionality of this habitat is persistently and increasingly jeopardized by anthropogenic pollution.^39,40^ For example, the Louisiana Department of Environmental Quality’s (LDEQ) 2024 Water Quality Inventory indicated that of the 518 unique waterbody subsegments that were impaired for use, over half were attributable to fecal contamination, including 4,300 miles of river, 27,600 acres of lake, 7,400 acres of estuary, and 372,000 acres of wetland.^41^ One potential source of such fecal pollution, OWTS, including both septic systems and ATUs, are managed by the Louisiana Department of Health (LDH) under its Onsite Wastewater Program. LDH reports there are 475,299 OWTS permitted for operation in Louisiana as of August 2023, 90% of which are ATUs, treating roughly 54.7 billion gallons of wastewater each year.^42^ However, metadata on the spatial distribution, functionality, or other characteristics of these systems are not publicly available.

### Estimating the Number of ATUs in Coastal Louisiana

Assuming each OWTS serves one household (a conservative assumption) and with the average Louisiana household size of 2.5 people, at least 25.5% of the state’s population would be served by onsite treatment, with 23% served by ATUs. ATUs provide biological treatment of wastewater through mechanical aeration in an aerobic chamber, reducing pollutant loadings via biodegradation prior to discharge. To estimate the total number of ATUs in each of Louisiana’s 64 parishes, we combined two unique data sets. First, we extracted the number of ATUs permitted by LDH annually in each parish from 1980 to 2016 as reported in a 2016 Water Environment Federation conference paper.^19^ Next, we extracted the number of total housing unit authorizations for each Louisiana parish from 1990 to 2023 (the entire available record) from the U.S. Census Bureau’s Building Permits Survey Time Series and Table Tool.^43^ Using the permitted ATU and permitted housing unit data sets, we calculated the permitted ATU/housing unit ratio (i.e., the ATU utilization rate, ATU-UR) for the state of Louisiana and for each individual parish annually from 1990 to 2016. In cases where permitted ATU or housing data were not reported, the year and parish entry were omitted in the calculation. We hypothesized that ATU/housing unit ratios would be distinct between metropolitan versus nonmetropolitan parishes, so we also used the 2023 Rural-Urban Continuum Codes (RUCC) to stratify parishes into groups based on RUCC.^44^ For each year and RUCC, we estimated the ATU-UR by summing the total number of permitted ATUs in all parishes within a RUCC value in a given year and dividing by the total number of housing units for the same. To project the number of permitted ATUs in each parish from 2017 to 2023, we multiplied the number of authorized housing units each year by the mean ATU/housing unit ratio (and the 95% confidence interval) observed from 1990 to 2015 for the RUCC group of which the parish was a member. We then estimated the cumulative number of permitted ATUs for the entire state by summing the annual number for each parish reported from 1990 to 2016 and projected per our calculations from 2017 to 2023. For each of the 24 coastal parishes (as defined in the 2023 Coastal Master Plan from the Coastal Protection and Restoration Authority^45^), we also estimated the cumulative number of permitted ATUs in the same manner. For both coastal parishes and the state of Louisiana, we also estimated the average annual ATU-UR from 1990 to 2016. Using the annual number and year of ATU permitting, we also estimated the average ATU age (as of 2023) for each for the 24 coastal parishes and the state.

### Estimating Pollutant Loadings Attributable to ATUs in Coastal Louisiana

To estimate the ATU-attributable loading of biodegradable organic matter (as measured via 5-day biochemical oxygen demand, BOD_5_), total suspended solids (TSS), total nitrogen (TN), and total phosphorus (TP) into coastal parish surface waters, we compiled typical effluent concentrations from the available literature. To characterize effluent concentrations from typical ATUs, we compiled reported data from five publications (Table S1)—two from Australia^46,47^ and three from the United States.^48–50^ For dysfunctional ATUs (i.e., without aeration), we assumed effluent quality would be comparable to septic tank effluent (STE) prior to biological treatment in a drain field. We extracted available STE data from eight studies (Table S2), including six from the United States^48–53^ and two from Australia.^46,47^ A pooled mean incorporating all data sets for each constituent was calculated as a sample size-weighted average. A pooled standard deviation was estimated by taking the square root of the sum of the variances, with each variance weighted by the study sample size less one, divided by the total sample size across all studies less the number of studies. For studies where the range was reported and the variance or standard deviation was not,^46,51,53^ the standard deviation was estimated as the range divided by four.^54^ For a study reporting the median, range, and sample size,^53^ the mean was estimated by summing the minimum, maximum, and two times the median and dividing the resulting value by four.^54^ In another study reporting the mean, 20th, and 80th percentiles,^47^ the standard deviation was estimated using the percentile values and Z-scores, assuming the data were normally distributed. Where the mean and coefficient of variation were reported,^48^ the standard deviation was calculated from the reported summary statistics. The mean daily loading from a functional ATU (L_F_, pounds per day) was calculated per eq 1, where C_ATU_ is the pooled mean for the constituent concentration (mg/L) estimated for typical ATU effluent, Q is the daily flow rate (gallons per day), and the coefficient provides unit conversion from milligrams per liter to pounds per gallon. The standard error about the mean was also calculated using eq 1 by substituting the standard error (estimated from the pooled constituent standard deviation and the total sample size) in for C_ATU_.

1 $$L_{F}=\left(8.3454\times 10^{-6}\right)\times C_{\mathrm{ATU}}\times Q$$

The daily loading from a dysfunctional ATU (L_DF_, pounds per day) was calculated similarly to eq 1, except C_ATU_ was replaced with C_STE_, where C_STE_ is the pooled mean or the standard error for the constituent concentration (mg/L) estimated for STE, which is representative of a dysfunctional ATU. In both cases, the daily discharge (Q, gallons per day) was calculated per eq 2,^55^ where N_P_ is the average household size and was obtained from U.S. Census Bureau data for each of the 24 coastal parishes.

2 $$Q=69.2+37.2\times N_{P}$$

The average total annual loading (TAL, pounds per year) attributable to all the ATUs in coastal parishes was calculated per eq 3, where T_ATU_ is the total number of ATUs, F is the functional proportion of ATUs (0 to 1.0), L_F_ is the daily loading for a functional ATU (pounds per day), L_DF_ is the daily loading for a dysfunctional ATU, and 365 is the coefficient for conversion from days to years. The 95% confidence interval about the mean was calculated using the combined standard errors for the functional and dysfunctional ATUs (square root of the sum of the squares) multiplied by 1.96 (z-score for 95% confidence interval assuming normality).

3 $$\mathrm{TAL}=\left[365\times T_{\mathrm{ATU}}\times F\times L_{F}\right]+\left[365\times T_{\mathrm{ATU}}\times \left(1-F\right)\times L_{\mathrm{STE}}\right]$$

### Onsite Wastewater Policy Analysis

To examine the content and structure of state and local OWTS policies, we drew on the policy design and implementation scholarship. Policy design and implementation are two interlinked but different facets of the policy process. Policy studies scholars emphasize the importance of ensuring that policy design choices and implementation tools align with the intended policy outcomes. Policy design focuses on the “substantive content” of public policies^56^ and contains significant information about the intended goals of actors, the characterization of target populations, and the value preferences of policymakers.^57,58^ Policy implementation includes, but is not limited to, choosing the appropriate tools, mechanisms, organizations, and local conditions.^59–61^ As such, implementation can be top-down, bottom-up, or a combination of the two.^62^ Even after broad policy goals and related policies are designed and adopted, implementing them requires careful consideration, necessitating local participation, involving a range of policy actors, and deploying the appropriate policy tools^56,61,63^ or policy mixes.^64^

### Policy Documents and Analysis

The core data used for the policy analysis are the sanitation codes of the state and two coastal parish governments – St. Tammany and Tangipahoa. We accessed the Louisiana state code from the state health department Web site^65^ and the parish ordinances were retrieved from the Municode Library.^66^ Based on the estimated number of ATUs, as previously described, we analyzed the two coastal parish governments with the largest number of permitted ATUs under their jurisdiction. Although the two parishes are adjacent to one another, they differ in terms of their economies, population, and government resources (Table S5).

We employed text analysis to investigate the extent to which the state and local sanitation codes adhere to the guidance in the EPA’s *Handbook for Managing Onsite and Clustered (Decentralized) Wastewater Treatment Systems* published in 2005.^9^ The EPA guidance is disaggregated into three stages of onsite wastewater management: (1) administration, (2) installation, and (3) operation and compliance. The three stages, in turn, comprise 13 program management elements, each with components at the basic, intermediate, and advanced levels. We used the description of each element at each level to create a dictionary of unique representative terms for OWTS management system characteristics. We then used the dictionary to identify relevant paragraphs in the sanitation codes and ordinances issued by the state and the two local governments. To maximize coverage, the dictionary includes appropriate wildcards (*) and Boolean combinations (OR/AND). We deployed the search process in the QDA Miner software (Version 6 2025, Provalis, Montreal, Canada) to conduct text analysis and iteratively refined the dictionary, ensuring that only relevant terms are included and minimizing any false positives. To reduce false positives, we limited the search results to include the terms wastewater, septic, sewage, or sewerage in the same paragraph. The ordinance documents vary significantly in length and choice of language, resulting in a wide variation in coverage of program elements as measured by individual mentions. Therefore, we converted the dictionary terminology frequency counts to binary measures, indicating the presence (1) or absence (0) of program elements at each level in the sanitary codes.

## Results and Discussion

### ATU Utilization and Abundance in Louisiana

As shown for each individual parish in Figures S1 – S4, annual housing unit data (1990 – 2023) and permitted ATU data (1990 – 2016) were compiled for all 64 parishes of Louisiana. Permitted ATU data were available for every parish except Plaquemines, and although housing data were available for every parish, some demonstrated many years with no reported new housing units (e.g., Bienville, De Soto, East Carroll), suggesting that in some cases the housing data are likely erroneous. The low number of ATUs permitted in 2016 compared to the historical average suggests the last year of the historical record is not reliable and was therefore excluded from the analysis. Despite anomalies in the annual data in certain parishes on certain years, as shown in Figure S5, the total number of annual housing units and permitted ATUs in Louisiana were strongly correlated from 1990 to 2015 with a Pearson correlation coefficient, r = 0.902 (95% CI: 0.790–0.955, *p* < 0.0001). With the exclusion of the 2016 data, annual housing units versus permitted ATUs in Louisiana were fit by a line (R^2^ = 0.8129) of slope, m = 0.677 (95% CI: 0.540–0.814), indicating an increase of 0.677 ATUs per unit increase in housing over the entire period. On an annualized basis (Figure S6), the average ATU-UR statewide from 1990 to 2015 was 0.737 (95% CI: 0.676–0.797), with a maximum annual value of 1.03 (2010). A value greater than 1.00 indicates that each housing unit resulted in the permitting of more than one ATU, likely due to new non-residential properties.

When considered on the rural–urban continuum, as indicated by RUCC, average annual ATU-URs in Louisiana increased with increasing rurality (Figure S7), although the trend was not strictly monotonic. As shown in Table S4, ATU-URs were significantly different (*p* < 0.0001, Kruskal–Wallis) between metropolitan (RUCC1-3) versus nonmetropolitan (RUCC4-9) parishes, with RUCC 1 and RUCC 2 and RUCC 8 and RUCC 9 parishes demonstrating significantly different utilization rates than all other RUCC groups, suggesting extreme differences for the most metropolitan and least metropolitan parishes. RUCCs 8 and 9 exhibited extreme annual values of permitted ATUs per housing unit with mean values of 23.1 (95%CI: 14.1–32.1) and 5.65 (95%CI: 3.77–7.53), respectively. Notably, the historic data that we have analyzed are for new ATU “tags” only and therefore do not include any maintenance permitting. Within RUCC 8 (8 parishes), this could be due to erroneous housing data in many years, with zero new housing units reported in U.S. Census data despite the LDH permitting of an average of 68.4 ATUs/year in RUCC 8 parishes. Similarly, in RUCC 9 (3 parishes), Claiborne parish reported zero housing units in many years. It is also possible that many existing homes on legacy systems upgraded to ATUs, which would require a new ATU permit without a new housing one. The erroneous housing unit data from these more rural parishes likely lead to inflated ATU/housing unit ratios. Nonetheless, across Louisiana, only RUCC 1 and RUCC 2 parishes have mean ATU/housing unit ratios less than one, implying that on average in 36 parishes the construction of one housing unit leads to the permitting of at least 1 ATU. Permitted ATU-to-housing unit ratios did not exhibit discernible temporal trends from 1990 to 2015 (Figure S9).

The concept of onsite system utilization rate has been previously elaborated to estimate the proportion of new housing units with an OWTS using national-level surveys conducted annually from 2015 to 2018.^5^ These surveys suggested national utilization rates between 29% and 32%, although seven states (Oklahoma, Idaho, Alabama, Kentucky, West Virginia, Vermont, and Michigan) reported utilization rates exceeding 50% over all four years. Although Louisiana was not included in this list, our analysis suggests that the statewide ATU utilization rate was 73.7% from 1990 to 2015. We also found evidence of large variations in the ATU utilization rate between parishes in Louisiana. In periurban parishes (RUCC 3 and 4), which account for 20% of newly constructed housing units, ATU utilization rates exceeded 100%. Utilization rates over 100% are likely explained by the codevelopment of nonresidential facilities such as businesses and schools that are also served by permitted ATUs. Conversely, in more urban settings (RUCC 1 and 2), where 76% of new housing was constructed over the same time frame, ATU utilization rates were 12% and 60%, respectively. Traditionally, an OWTS is conceptualized as a rural low-density paradigm. However, our findings emphasize the growing importance of peri-urban spaces as venues for OWTS deployment. In these spaces at the periphery of municipalities and their infrastructure, OWTS may provide planners and developers with an efficient way to continue development and expansion without the need to incur the large capital costs associated with centralized sewer system expansion.^67^ While this mode of development is typically more considered for the provision of safe sanitation in low- and middle-income countries,^68^ we find evidence that even in a high-income country such as the United States, the flexibility and efficiency provided by decentralized wastewater treatment could lead to increasing deployment even in spaces historically considered to be appropriate for centralized sewer systems. In Louisiana, high ATU utilization rates combined with the development of large numbers of housing units in peri-urban settings will lead to hyperdense OWTS zones.^19^ In the event these zones are also characterized by high rates of treatment dysfunction, the ability of the surrounding environment to attenuate the impact could be much more limited.

Using the ATU/housing unit ratio for each RUCC group, we projected a total of 412,552 permitted ATUs (95%CI: 400,125–424,928) were in Louisiana by the end of 2023—a 28% increase over 7 years (Figure S8). Our estimate suggests 87% of OWTS permitted in Louisiana are ATUs, which is only slightly lower than the 90% proportion previously reported by LDH.^42^ Importantly, our approach enables us to further consider the spatial distribution of ATUs by parish and region, which has not been previously feasible with publicly available data. As shown in Figure 1, roughly 58% of the permitted ATUs (237,809/412,552) are within the 24 coastal parishes, with the greatest numbers in St. Tammany (32,570) and Tangipahoa (30,462) followed by Calcasieu (23,996) and Lafayette (21,206). Despite having similar numbers of ATUs, the ATU utilization rate for Tangipahoa (1.03, Table S3) is more than double the rate in St. Tammany (0.491) directly to the east. This is likely attributable to the larger municipal sewage service areas in several cities in St. Tammany compared to the much smaller municipal footprints in St. Tammany. Notably, there was no significant difference in the ATU-UR between coastal and noncoastal parishes (*p* > 0.9999, Kruskal–Wallis). The highest ATU/housing unit ratio among coastal parishes was Assumption Parish with 2.48 ATUs per housing unit, which also has the oldest ATUs on average at 21.4 years. The youngest average age of 4.2 years is estimated for Orleans parish with only 1,100 ATUs and the lowest ATU-UR of 0.037. The average age of an ATU in the coastal parishes is 14.92 years, which is comparable to the average age of 15.25 years for all of Louisiana.

**Figure 1:**
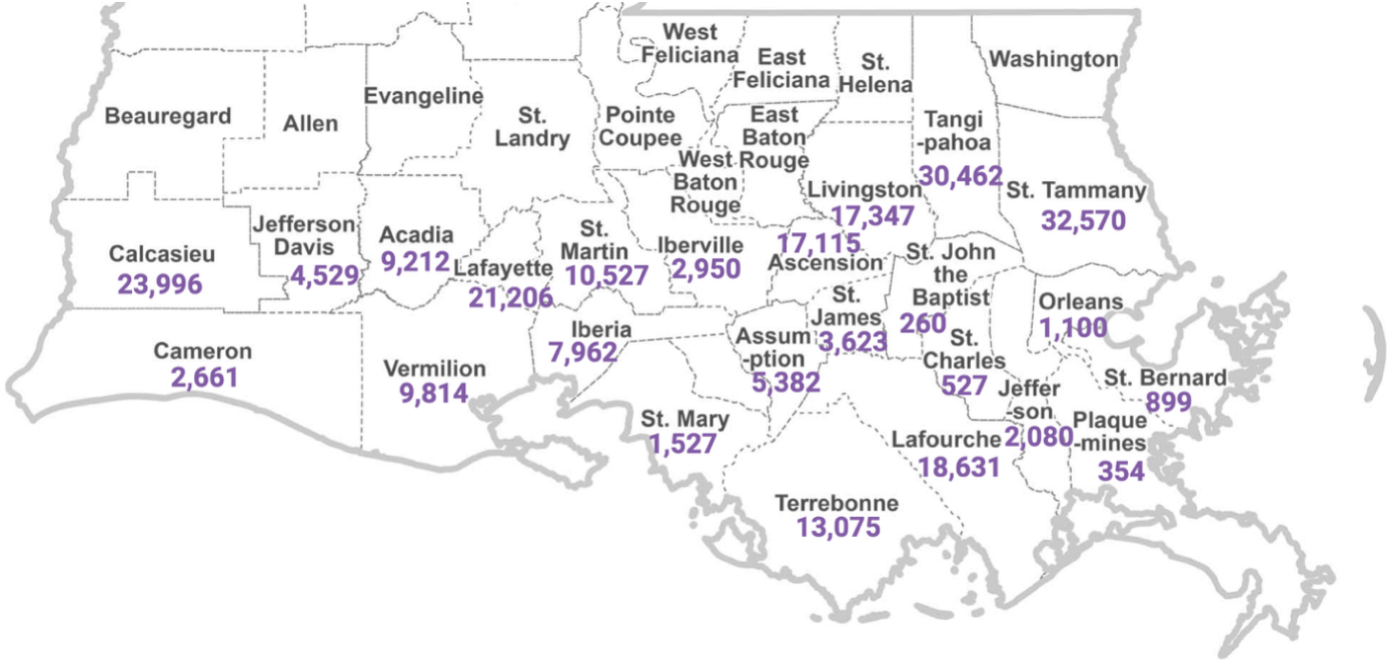
Estimated number of permitted ATUs in each of the 24 coastal parishes of Louisiana as of 2023.

Historically, OWTS have been acknowledged among leading sources of near-shore ocean pollution, although much of the research has focused on traditional septic systems.^69^ While much less is known about the role of ATUs, the large number we estimate to be in the coastal parishes of Louisiana and their direct discharge to surface waters suggests the need for further consideration. For instance, the number of permitted ATUs in St. Tammany and Tangipahoa suggests an average density of 38 ATUs per square mile. On an annual basis, these ATUs would discharge roughly 1.0 million gallons of effluent per square mile into surface waters that are tributary to Lake Pontchartrain, the second-largest estuary in the continental United States.^36^ In the coastal region, we also find spatial differences in ATU-UR persist with utilization rates greater than 100% in half of coastal parishes. This suggests the potential for high-density ATU utilization in coastal watersheds. Additionally, we find the average age of a permitted ATU in the coastal parishes to be roughly 15 years as of 2023. Age is a known risk factor for OWTS dysfunction, as failure rates typically increase with system age.^67^ While the service life of a septic tank can be 15 to 40 years,^70^ much less is known about ATU service life, particularly in coastal settings. Given the use of mechanical aeration for biological treatment, the service life for an ATU is likely to be strongly dependent on routine maintenance, although roughly half of homeowners report not maintaining their OWTS during surveys.^71^ In coastal Louisiana, high ATU utilization rates, aging systems, lack of routine maintenance, and direct surface water discharge create the opportunity for systemic water quality degradation.

### Potential ATU Pollutant Loadings to Coastal Surface Waters

The estimated daily discharge from all ATUs in the 24 coastal parishes is 39.4 million gallons per day (MGD) or 14.4 billion gallons per year (BGY), which is roughly 90% of the annual discharge from the South Wastewater Treatment Plant (WWTP) serving Baton Rouge, Louisiana.^72^ For a typical functional ATU, the average BOD_5_, TSS, TN, and TP concentrations (mg/L) expected in the effluent are tabulated in Table S1. In the case of a dysfunctional ATU, as approximated by STE, the expected concentrations of the same constituents are summarized in Table S2. These values suggest that ATU dysfunction would be associated with 10.2-, 2.8-, and 2.0-fold increases in discharged BOD_5_, TSS, and TN, respectively. Conversely, the pooled data indicate that the TP concentration would be 50% less for a dysfunctional ATU compared to a functional one. Given the annual discharge from all ATUs in the coastal parishes and a functional proportion (0 to 100%), we estimated the total loading for four different pollutants, as shown in Table 1. Previous inspection programs suggest roughly 40% of ATUs are functional (i.e., aerator functioning properly), leading to an estimated annual loading of 16.9 million pounds of BOD_5_, 8.83 million pounds of TSS, 7.51 million pounds of TN, and 2.18 million pounds of TP into coastal surface waters from ATUs. These loadings equate to 260% more BOD_5_, 40% more TSS, 70% more nitrogen, and 220% more phosphorus than is discharged annually from the wastewater treatment plants serving Baton Rouge (South) and New Orleans (East Bank and West Bank) combined.^72^ If ATU functionality were increased from 40% to 90%, then BOD_5_, TSS, and TN loadings could be reduced by 70.5%, 42.8%, and 32%, respectively. In the case of TP, the data suggest that functional ATUs would discharge more phosphorus than is measured in the STE. However, it should be noted that measurements of phosphorus in ATU effluent are extremely limited (n = 67), with a study from Australia (2010, n = 36) indicating the concentration is comparable to STE,^46^ while a study from the United States (1976, n = 31) found double the concentration in ATU effluent.^48^ It is possible that the anaerobic conditions, along with primarily settling in a septic tank, could promote higher phosphorus removal compared to an aerobic suspended growth chamber with less efficient biomass settling, although a 100% increase, as observed in the 1976 study, seems unlikely. In general, across all parameters, there is a great need for effluent data from ATUs in use at households in the United States, with most of the data originating from Australia, and the overall sample sizes being about half of those for STE.

**Table 1:** Total Pollutant Loadings to Surface Waters in the Coastal Parishes Attributable to ATU Discharges

Proportion of ATUs Functional	BOD Loading 10^6^ lb./yr. Mean (95% CI)	TSS Loading 10^6^ lb./yr. Mean (95% CI)	TN Loading 10^6^ lb./yr. Mean (95% CI)	TP Loading 10^6^ lb./yr. Mean (95% CI)
100%	2.59 (2.24–2.95)	4.30 (4.04–4.55)	4.62 (3.94–5.31)	3.18 (2.65–3.72)
90%	4.98 (4.64–5.32)	5.05 (4.77–5.34)	5.10 (4.49–5.72)	3.02 (2.53–3.50)
80%	7.36 (7.00–7.72)	5.81 (5.41–6.21)	5.59 (5.03–6.14)	2.85 (2.42–3.28)
70%	9.74 (9.33–10.2)	6.57 (6.03–7.10)	6.07 (5.56–6.57)	2.68 (2.31–3.06)
60%	12.1 (11.6–12.6)	7.32 (6.63–8.02)	6.55 (6.09–7.01)	2.51 (2.19–2.84)
50%	14.5 (13.9–15.1)	8.08 (7.22–8.93)	7.03 (6.60–7.46)	2.35 (2.08–2.62)
**40%**	**16.9** **(16.2–17.6)**	**8.83** **(7.81–9.85)**	**7.51** **(7.10–7.92)**	**2.18** **(1.96–2.40)**
30%	19.3 (18.5–20.0)	9.59 (8.40–10.8)	7.99 (7.58–8.41)	2.01 (1.84–2.18)
20%	21.7 (20.8–22.5)	10.3 (8.98–11.7)	8.48 (8.05–8.91)	1.85 (1.72–1.97)
10%	24.0 (23.1–25.0)	11.1 (9.57–12.6)	8.96 (8.49–9.42)	1.68 (1.59–1.77)
0%	26.4 (25.3–27.5)	11.9 (10.2–13.5)	9.44 (8.93–9.95)	1.51 (1.43–1.60)

Estimates of the water quality impacts directly attributable to OWTSs are largely constrained to septic tanks in small-scale watershed or subwatershed studies.^67,73–75^ In the case of nutrients, for example, in Ohio, failing onsite wastewater systems have been estimated to contribute 3% to 7% of the total nitrogen (TN) and 3% to 9% of the total phosphorus (TP) across the Great Miami, Scioto River, and Muskingum River watersheds.^76^ Systems-level analyses of water quality impacts across entire states or regions and for mechanical systems such as ATUs are rare. In this case, our estimated ATU pollutant loadings across only the 24 coastal parishes of Louisiana would be equivalent to 48% of the TN and 84% of the TP discharged from all the major publicly owned treatment works (POTWs) in Louisiana in 2020, as reported by the Hypoxia Task Force.^76^ Nonetheless, efforts to address nutrient pollution from OWTSs, septic tanks specifically, are only implemented on a state-by-state basis, with only Ohio and Arkansas reporting programs in the 2023 Hypoxia Task Force Report to Congress.^76^ OWTS, and particularly ATUs discharging to surface waters in coastal regions, afford a compelling opportunity to reduce nutrient loadings into the Gulf.

### OWTS Policy in Coastal Louisiana

Our water quality analysis reveals the likely systemic degradation of Louisiana’s coastal surface waters resulting from the intersection of high ATU utilization in peri-urban coastal parishes combined with widespread dysfunction. To better understand the policy mechanisms related to these outcomes, we performed comparative text analysis of policy documents at the state level (Louisiana Sanitary Code) and local levels (Parish Ordinances) for the two coastal parishes with the greatest number of ATUs—St. Tammany and Tangipahoa, which also happen to be adjacent to one another on the north shore of Lake Pontchartrain. Per the EPA Handbook, policy designed to the basic level implies low relative risks for environmental sensitivity, public health, treatment complexity, and wastewater characteristics;^9^ however, as we have described previously, the unique geographical location of Louisiana relative to the hypoxic zone, the high ATU utilization rate, and the use of more complex mechanical treatment processes may benefit from program design that accounts for increasing relative risks. Nonetheless, across these documents, as shown in Figure 2, we find the state and local onsite wastewater management programs are most aligned with the “basic” level. Fewer intermediate-level elements are incorporated across all three jurisdictions, and none of the advanced-level elements are reflected in the state and local sanitation codes.

**Figure 2:**
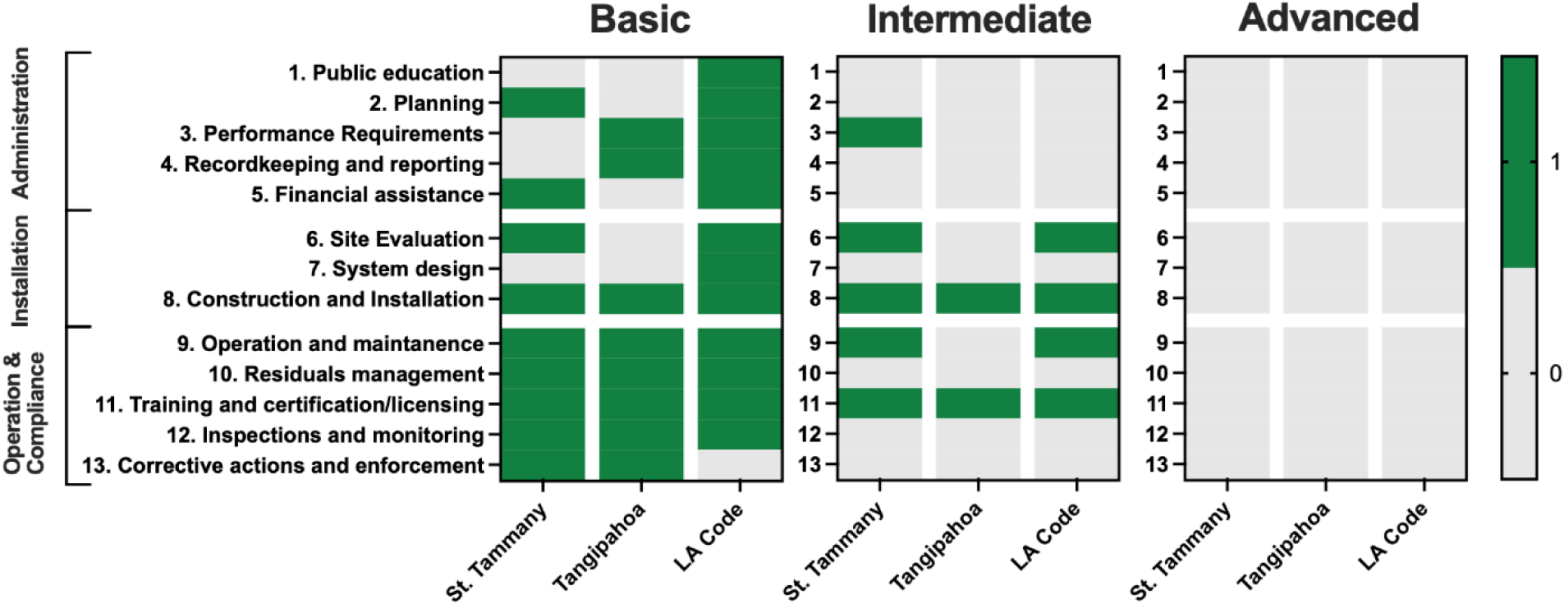
Coverage of 13 program elements for OWTS management at the basic, intermediate, and advanced levels in St. Tammany and Tangipahoa ordinances, and the Louisiana Sanitary Code.

The state code provides the most comprehensive coverage, with 12 of the 13 basic design elements incorporated in the sanitation code. The two local jurisdictions also reflect significant coverage, with each incorporating eight elements. The state and local policy design largely pertains to operation and compliance (elements 9–13), followed by administration (elements 1–5) and installation (elements 6–8). Within the operation and compliance elements, policy design emphasizes licensing of manufacturers, installers, and maintenance providers, handling of residuals by licensed haulers, and installation inspections by state health officers. However, consistent with the basic level, the policy design largely centers on activities prior and up to completed installation but does not incorporate ongoing and routine maintenance beyond an initial two-year service contract provided with the purchase of a new ATU. This is typical of OWTS governance across many settings, with anachronistic “set it and forget it” paradigms prevailing despite the existence of technologies that could enable a more preventative approach. Notably, beyond the initial two-year period, the responsibility for compliance lies largely with the property owner, with ambiguous procedural guidance. The policy documents do not extensively codify administrative program elements, such as public education, planning, performance requirements, and record-keeping, beyond the basic level. As a result, while the policy design might reflect many of the program elements, the state and local ordinances do not adequately incorporate the necessary policy tools to support policy implementation.^77^ This gap highlights the importance of incorporating the correct tools to implement the policy design. Overall, the policy design indicates a hybrid management model premised on both a maintenance contract and homeowner awareness.

The text analysis identified three gaps in management program elements. First, despite a management model premised on homeowner awareness, public education elements are present only in state-level policies at the basic level. This reveals a possible mismatch between the governmental entities responsible for homeowner education (i.e., local governments) and the level at which policy is codified (i.e., state level). The second gap relates to the absence of enforcement and punitive mechanisms in the state’s code. While the two local government ordinances include this program element at the basic level, neither has adopted elements related to monitoring and corrective actions, as incorporated in their sanitation codes, at the intermediate or advanced levels. As a result, while the state code covers many of the basic elements of wastewater management systems, if homeowners and other parties responsible for long-term maintenance fail to meet the required standard, then there are limited options for ongoing corrective action beyond reactive nuisance reporting.^78^ This gap, combined with the relatively infrequent mention of monitoring-related activities, indicates a lack of ongoing mechanisms to implement, manage, and enforce onsite wastewater treatment management policy. Finally, the third gap relates to the absence of financial resources to remediate dysfunctional systems, a burden that largely falls on the individual homeowner. Although federal programs are available to provide grants and low-interest loans for residential OWTS repairs, the policy design suggests that local and state governments may not have the capacity to effectively liaise between individual homeowners and federal resources. The administrative burden of accessing federal funds is a known barrier to sustaining functional onsite wastewater treatment systems.^27^ Although the case-based analysis in this study limits the generalizability of the findings, we also note some differences in the policy outputs based on local resources and capacity. The local government with higher capacity, as measured by income levels and total general fund revenues reported in the last financial year^79,80^ (St. Tammany Parish), is associated with a wider coverage of design and implementation elements at the intermediate level. This variation may reflect the impact of local capacity on designing policy and implementing the appropriate tools to achieve policy goals.

Together, our ATU spatial analysis, pollutant loading assessment, and policy text analyses indicate that ATUs are being utilized at rates well above the national average throughout Louisiana, with particularly high densities possible in peri-urban settings near coastal surface waters. Limited compliance monitoring, enforcement, and public awareness likely drive the proliferation of dysfunctional ATUs, as indicated by sporadic inspection programs. We simultaneously estimate that widespread ATU dysfunction is sufficient to cause systemic water quality degradation in coastal surface waters. While protecting water quality is vital to the culture and economy of Louisiana,^81^ limited financial resources at the household and parish levels hamper the ability to remediate ATU dysfunction at scale.^82^

In response, there is a clear need to pioneer innovative solutions in policy design, implementation, and financing to better reflect the economic value delivered by functional onsite wastewater systems that avert systemic water quality degradation. For example, recently, St. Tammany Parish successfully passed an ordinance requiring the inspection of ATUs that discharge off property every three years.^83^ Interestingly, the inspection program is being funded by the St. Tammany Mosquito Abatement District^84^ because surface waters contaminated with sewage provide an ideal habitat for the proliferation of mosquito species that transmit West Nile virus.^85^ Here, we briefly consider the potential of market-based solutions to fund ATU maintenance programs.

### Potential to Deploy Market-Based Solutions

Generating sustainable funding streams for proactive OWTS governance requires creative deployments of financial tools. To demonstrate the potential of such approaches, we explored enhanced ATU maintenance and nutrient removal through an abridged benefit-cost analysis (BCA) and a novel financing mechanism, an Environmental Impact Bond (EIB). Our BCA demonstrates that nutrient recovery and operational optimization of aerobic treatment units (ATUs) in coastal Louisiana can generate tangible economic and environmental gains. Assuming a 300-gallon-per-day treatment rate and conservative phosphate recovery (60%), each ATU could recover roughly 8.33 kg/year of struvite, worth about USD $9.84/year per ATU at present-day prices.^86,87^ Although the financial benefit of struvite recovery is less than $1 per month at present, volatility in fertilizer prices and strong interest in resource recovery from decentralized wastewater systems justify its consideration.^88^ Furthermore, we estimate optimization of the ATU aeration can reduce electricity usage by at least 30% under aerobic operation.^89^ Assuming an ATU power consumption of ∼80W (700 kWh/yr) and Louisiana electric prices of $0.12/kWh, we calculate a conservative estimated savings of ∼$25/year per ATU. The total of $34.84/year per ATU value (∼$11 million/year over all ATUs in Louisiana) is roughly equivalent to the estimated cost for an ATU maintenance program funded through property taxes for St. Tammany Parish during a recent referendum ($100 every three years), indicating likely positive net present values from both nutrient recovery and cost savings alone.^82^

However, BCA analysis demonstrates only economic feasibility. Turning economic benefits into action requires financing, which is challenging for ATUs due to the multijurisdictional regulatory environment, disperse benefits distributed to multiple stakeholders, and because local municipalities are often capital-constrained.^90^ Environmental impact bonds (EIBs) offer a systems-based financing mechanism to catalyze implementation. EIBs link private investment returns to verified environmental performance, aligning fiscal and ecological outcomes.^91^ In this model, investors provide upfront capital to parishes for ATU retrofits and nutrient recovery, and repayments are tied to measurable reductions in nutrient loading, energy use, and operating costs. Such performance-based financing has been successfully deployed for watershed and wetland restoration, wildfire mitigation, and sediment management and can similarly be applied to decentralized wastewater infrastructure.^91–94^ By internalizing environmental benefits within a circular funding model, EIBs offer a pathway to consolidate ATU management and stakeholder under one fiscal structure for accelerating change.

Our analysis of the complex sociotechnical aspects of OWTS, particularly of advanced ATUs, which are understudied compared to traditional septic tanks, is geographically constrained to Louisiana owing to the unique data sets required. While the specifics of our findings may not be generalizable to all OWTSs in all states, the themes are likely typical. What is unique is our ability to estimate the systemic water quality impacts of widespread ATU utilization and dysfunction by combining effluent data with estimated ATU numbers across Louisiana. We find that the nutrient loadings from ATUs into coastal waterways are on the order of those associated with municipal WWTPs and yet are overlooked in national efforts to reduce the size of the hypoxic zone in the Gulf. Despite the proliferation of ATUs and the high likelihood of adverse water quality impacts, management efforts remain at basic levels, with limited governance owing to resource constraints. As we have found here, the status quo paradigms of onsite wastewater treatment, throughout the United States and elsewhere, have the potential to jeopardize water quality in watersheds at all scales.

## Supplementary Material



## Notes

ATU effluent data from literature; septic tank effluent data from literature; total housing units and permitted ATUs in individual Louisiana parishes; annual housing units and permitted ATUs in Louisiana (1990–2016); permitted ATU-to-housing unit ratio in Louisiana (1990–2016); permitted ATU-to-housing unit ratios stratified by RUCC; cumulative number of ATUs in Louisiana (1990–2023); cumulative number of ATUs, ATU utilization rate, and mean age in Louisiana parishes; demographic and governance statistics for St. Tammany and Tangipahoa parishes (PDF)
