# Supplementary material for: Aerobic Treatment Units for Widespread Onsite Wastewater Treatment in Coastal Louisiana: Pollution, Policy, and Market-Based Solutions
Source: Environ Sci Technol. 2026 May 14;60(20):14307–17. doi: 10.1021/acs.est.5c17284 (PMC13217556; doi:10.1021/acs.est.5c17284)
Supplement: Supplementary file 1 [file es5c17284_si_001.pdf]

Supporting Information: Aerobic Treatment Units for Widespread On-Site Wastewater Treatment in Coastal Louisiana: Pollution, Policy, and Market-Based Solutions

Anmol Soni[1], Banna Das[2], Matthew Brand[2], Aaron Bivins[2*]

[1]Department of Public Administration, Louisiana State University, Baton Rouge, LA, 70803, USA
[2]Department of Civil & Environmental Engineering, Louisiana State University, Baton Rouge, LA, 70803, USA
*3255 Patrick F. Taylor Hall, Baton Rouge, LA, 70803, USA; abivins@lsu.edu

Table S1. ATU effluent data extracted from literature
Table S2. Septic tank effluent data extracted from literature
Figure S1. Total Housing Units and Permitted ATUs in 16 Parishes of Louisiana
Figure S2. Total Housing Units and Permitted ATUs in 16 Parishes of Louisiana
Figure S3. Total Housing Units and Permitted ATUs in 16 Parishes of Louisiana
Figure S4. Total Housing Units and Permitted ATUs in 16 Parishes of Louisiana
Figure S5. Annual total housing units versus permitted ATUs in Louisiana
Figure S6. Annual permitted ATUs and housing units ratio from 1990 to 2016
Figure S7. Ratios of permitted ATUs to housing units by parish stratified by RUCC
Figure S8. Cumulative number of ATUs 1990 – 2023
Table S3. Coastal parish cumulative ATUs, ATU utilization rate, and mean age
Figure S9. ATU utilization rate from 1990 to 2016 in coastal parishes versus all Louisiana
Table S4. Permitted ATU/housing unit ratios for Louisiana parishes
Table S5. Demographic and governance statistics for St. Tammany and Tangipahoa

Table S1. ATU Effluent Data

Study	Location	BOD (Mean ± SD)	TSS (Mean ± SD)	TN (Mean ± SD)	TP (Mean ± SD)
Levett *et al.*[1]	Australia	30 ± 43<br>n = 31	98 ± 39.3<br>n = 31	39.6 ± 32.9;<br>n = 31	16.3 ± 7.7<br>n = 31
Otis and Boyle[2]	United States	47 ± 37.1<br>n = 63	53 ± 10.1<br>n = 69	37.6 ± 13.5<br>n = 38	35.2 ± 24.3<br>n= 36
Garcia *et al.*[3]	United States	1 ± 0.1<br>n = 7	2.7 ± 0.5<br>n = 7	not measured	not measured
Charles *et al.*[4]	Australia	11 ± 7.7<br>n = 140	18 ± 13.1<br>n = 141	not measured	not measured
Du *et al*. (October)[5]	United States	4.8 ± 2<br>n = 8	5.9 ± 2<br>n = 8	not measured	not measured
Du *et al*. (January)[5]	United States	10 ± 6<br>n = 8	18 ± 20<br>n = 8	not measured	not measured
**Pooled Estimate**		**21.6 ± 24.4<br>n = 257**	**35.8 ± 17.6<br>n = 264**	**38.5 ± 24.2<br>n = 69**	**26.5 ± 18.6<br>n = 67**

Table S2. Septic Tank Effluent Data

Study	Location	BOD (Mean ± SD)	TSS (Mean ± SD)	TN (Mean ± SD)	TP (Mean ± SD)
Levett *et al.*[1]	Australia	119 ± 52.8<br>n = 29	254 ± 430.3<br>n = 29	126 ± 101<br>n = 29	17.4 ± 14<br>n = 29
Otis and Boyle[2]	United States	198 ± 99<br>n = 94	54 ± 9.2<br>n = 93	55.3 ± 23.2<br>n = 53	14.6 ± 11.7<br>n= 54
Garcia *et al.*[3]	United States	76.4 ± 9.3<br>n = 7	90.12 ± 15<br>n = 7	not measured	not measured
Charles *et al.*[4]	Australia	224 ± 152.1<br>n = 43	379 ± 358.8<br>n = 43	160 ± 74.6<br>n = 45	21 ± 10.5<br>n = 46
Du *et al*. (October)[5]	United States	75 ± 20<br>n = 8	20 ± 7<br>n = 8	not measured	not measured
Du *et al*. (January)[5]	United States	60 ± 10<br>n = 8	27 ± 10<br>n = 8	not measured	not measured
Anderson *et al.*[6]	United States	93.5 ± 27.5<br>n = 11	not measured	44.2 ± 8.5<br>n = 11	8.6 ± 2.45<br>n = 11
Lowe et al.[7]	United States	327 ± 197<br>n = 59	85.5 ± 41.0<br>n = 61	68.0 ± 23.0<br>n = 61	13.2 ± 8.2<br>n = 61
Loomis et al.[8]	United States	240 ± 53<br>n = 214	49 ± 16<br>n = 208	62 ± 13<br>n = 168	10 ± 2<br>n = 237
**Pooled Estimate**		**220 ± 101<br>n = 473**	**98.7 ± 154<br>n = 457**	**78.6 ± 41.4<br>n = 367**	**12.6 ± 7.3<br>n = 438**

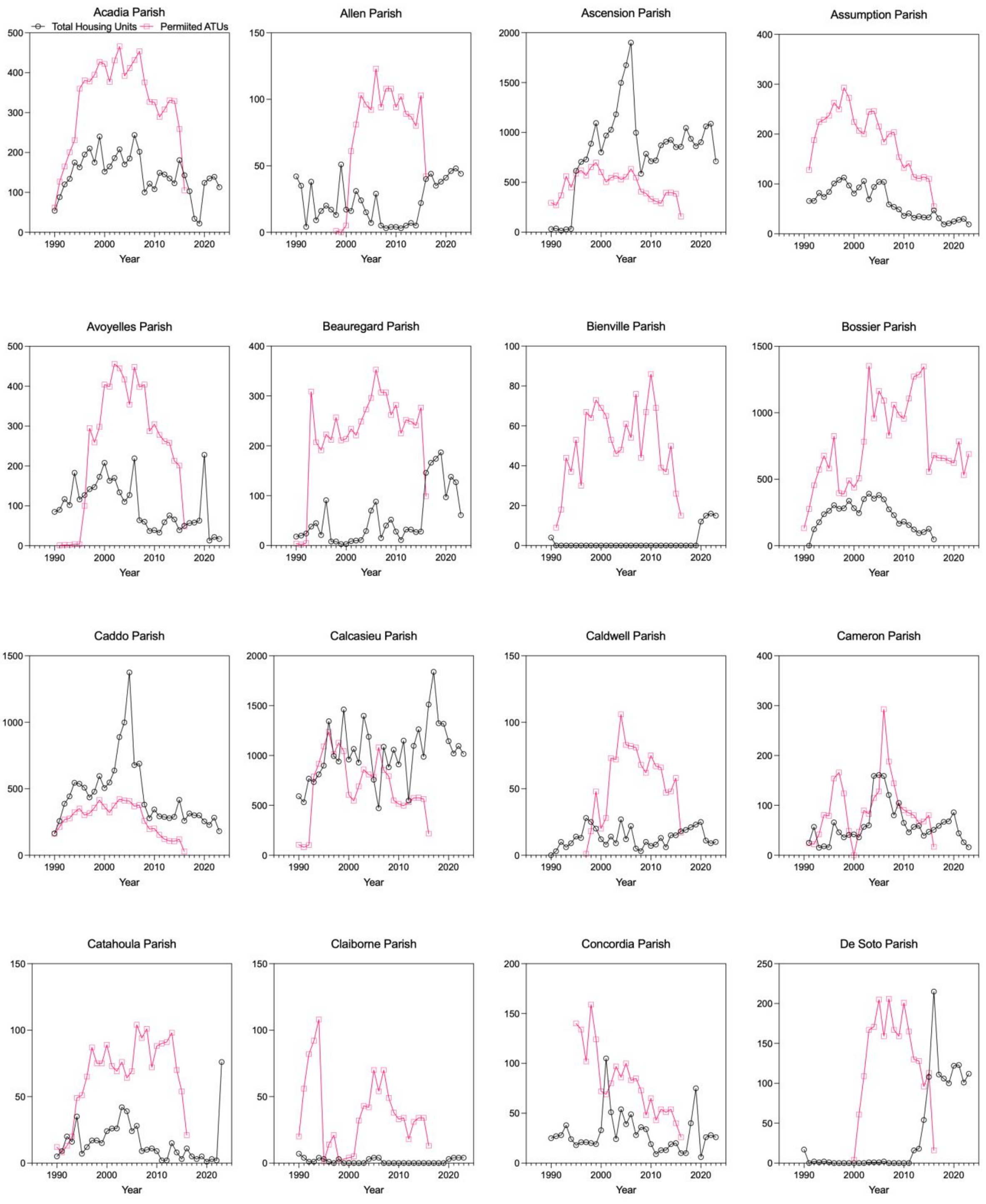

Figure S1. Total housing units and permitted ATUs from 1990 to 2016 in Acadia, Allen, Ascension, Assumption, Avoyelles, Beauregard, Bienville, Bossier, Caddo, Calcasieu, Caldwell, Cameron, Catahoula, Claiborne, Concordia, and De Soto Parishes, Louisiana.

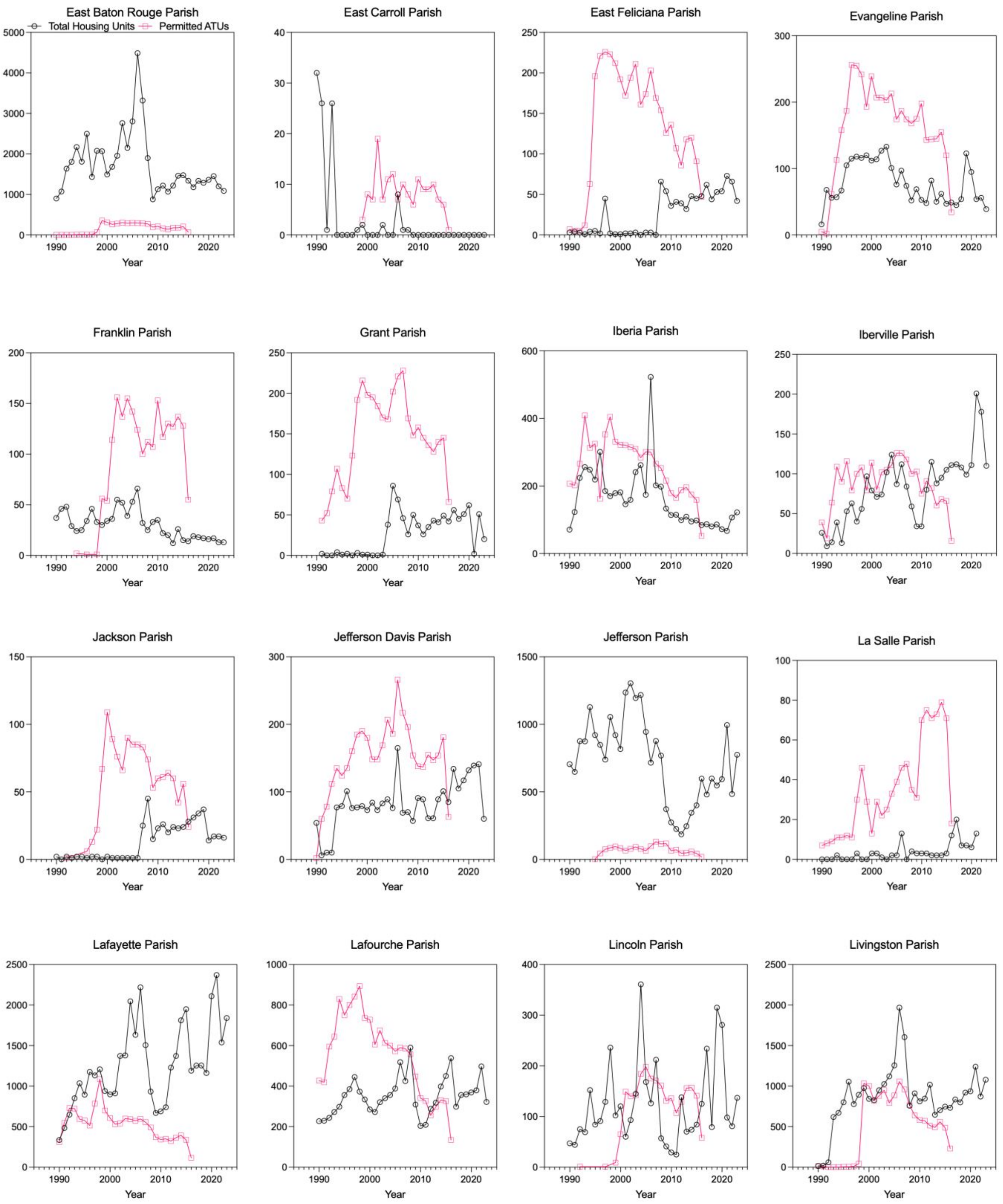

Figure S2. Total housing units and permitted ATUs from 1990 to 2016 in East Baton Rouge, East Carroll, East Feliciana, Evangeline, Franklin, Grant, Iberia, Iberville, Jackson, Jefferson Davis, Jefferson, La Salle, Lafayette, Lafourche, Lincoln, and Livingston Parishes, Louisiana.

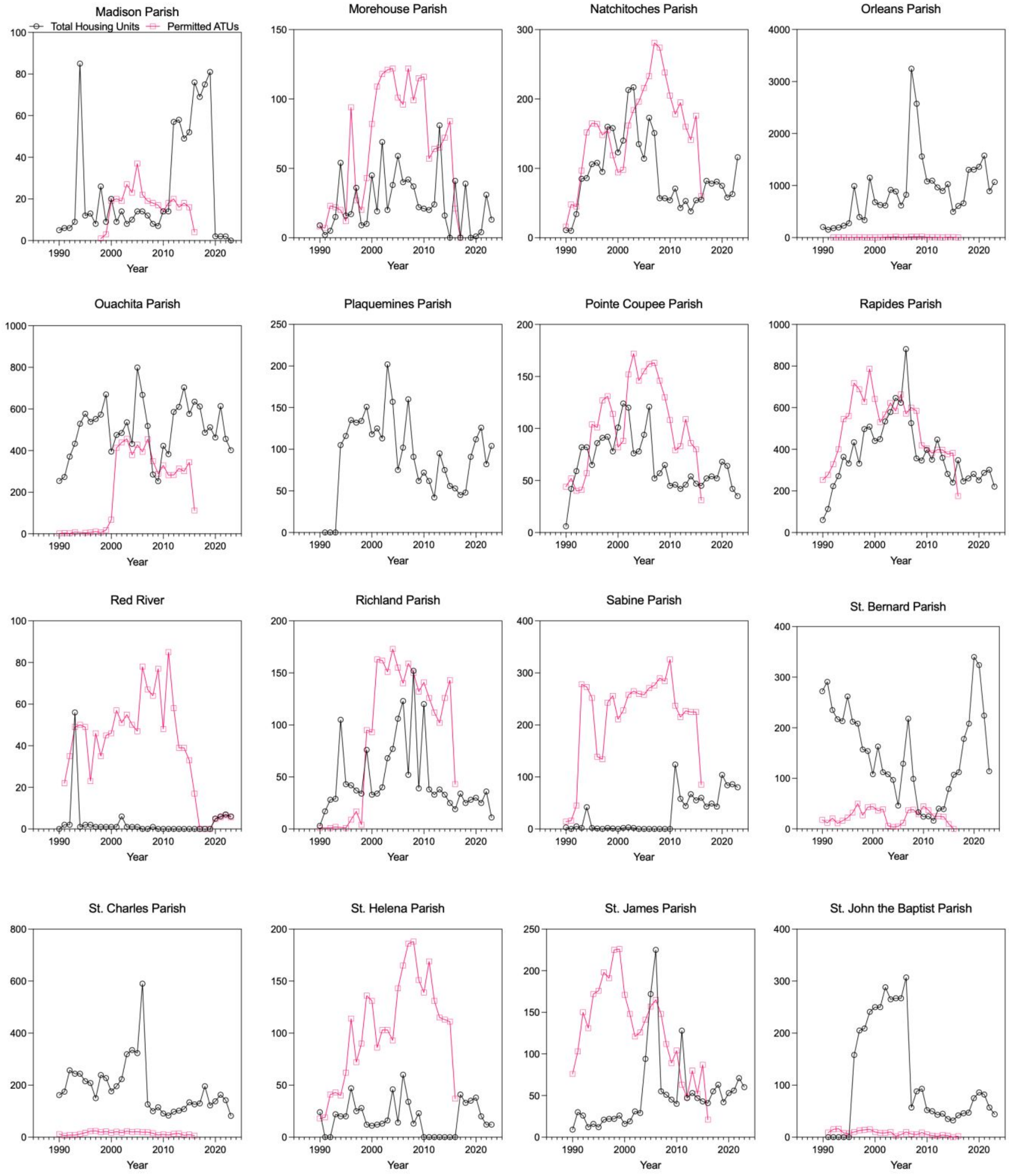

Figure S3. Total housing units and permitted ATUs from 1990 to 2016 in Madison, Morehouse, Natchitoches, Orleans, Ouachita, Plaquemines, Pointe Coupee, Rapides, Red River, Richland, Sabine, St. Bernard, St. Charles, St. Helena, St. James, and St. John the Baptist Parishes, Louisiana.

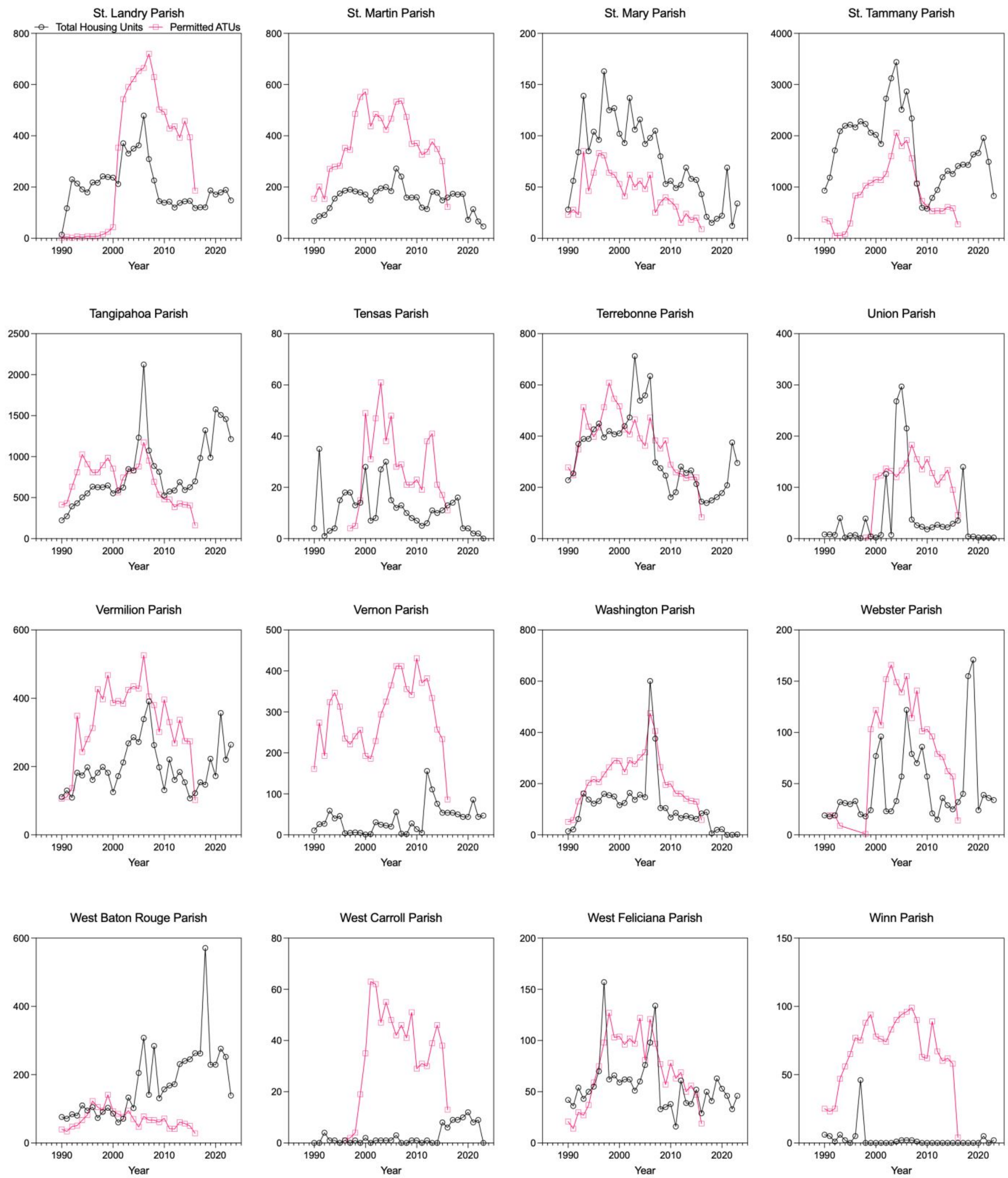

Figure S4. Total housing units and permitted ATUs from 1990 to 2016 in St. Landry, St. Martin, St. Mary, St. Tammany, Tangipahoa, Tensas, Terrebonne, Union, Vermillion, Vernon, Washington, Webster, West Baton Rouge, West Carroll, West Feliciana, and Winn Parishes, Louisiana.

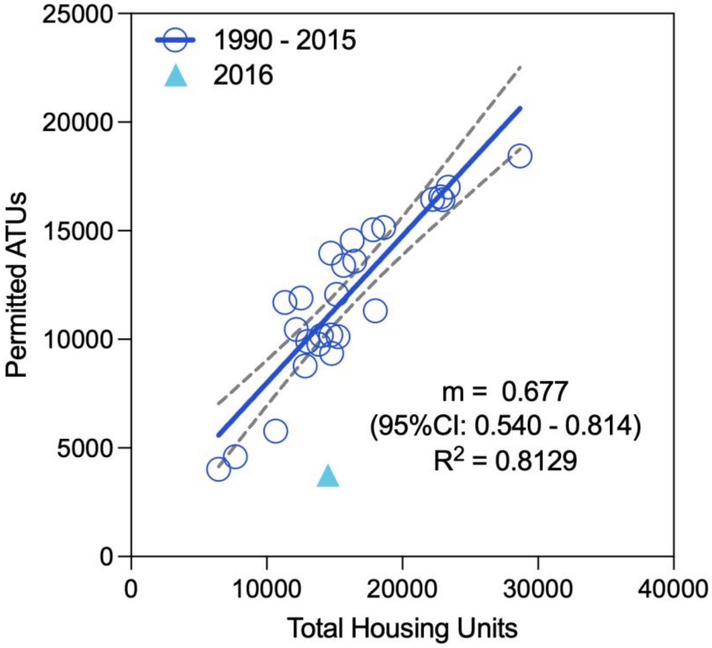

Figure S5. Annual total housing units (x) versus annual permitted ATUs in Louisiana from 1990 to 2016.

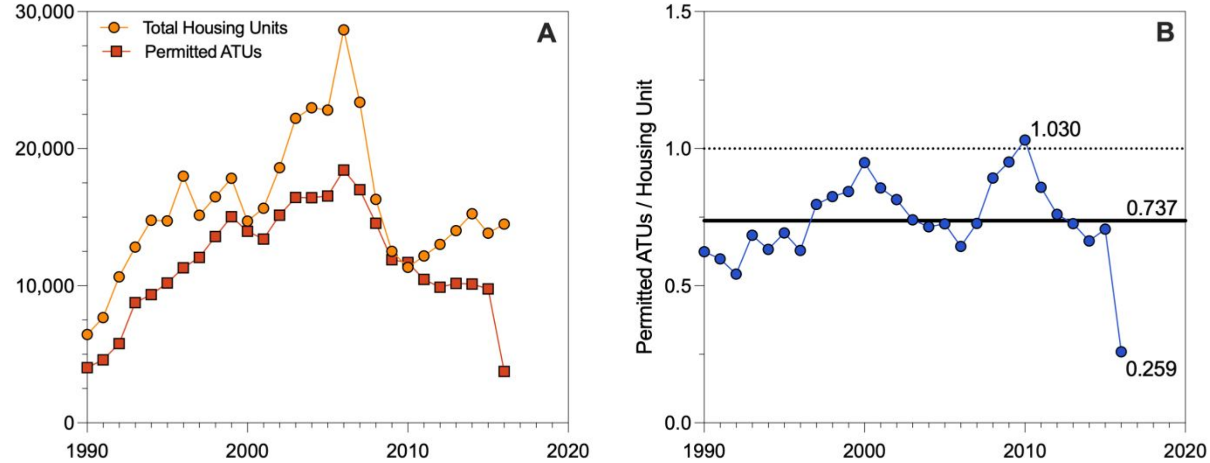

Figure S6. The annual number of permitted ATUs and housing units in the state of Louisiana from 1990 to 2016 (A) and the ratio of permitted ATUs to housing units (i.e., the ATU utilization rate) over the same period (B).

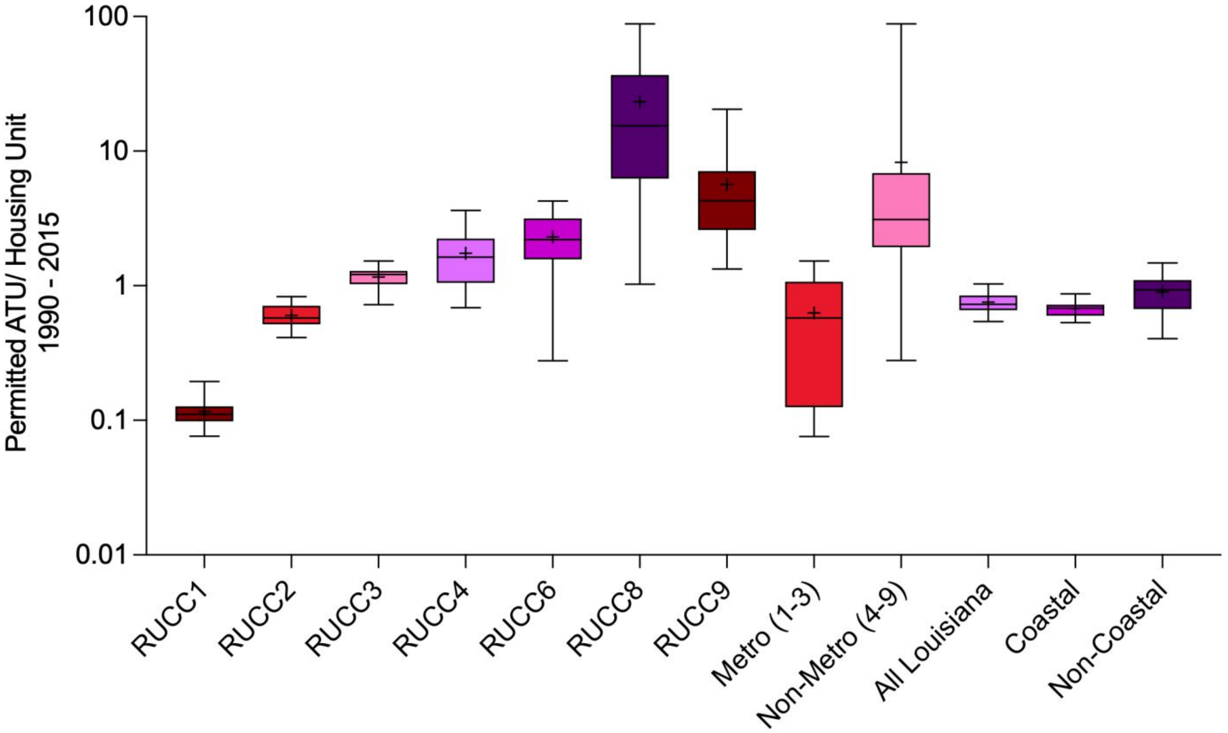

Figure S7. Ratios of permitted ATUs to housing units by parish stratified by rural-urban continuum codes (RUCC) and metro (RUCC1-3) versus non-metro (RUCC4-9) in 2015 (A) and 2016 (B) in Louisiana.

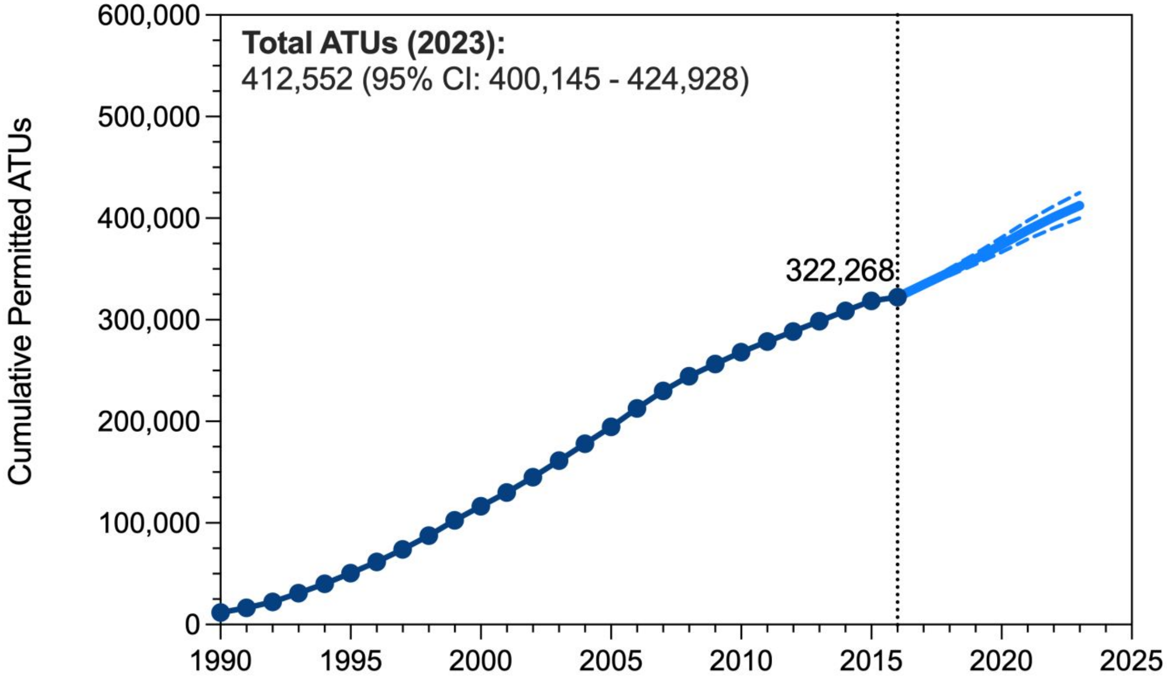

Figure S8. The cumulative number of permitted ATUs in Louisiana from 1990 to 2016 and projected from 2017 to 2023.

Table S3. Cumulative ATUs, ATU utilization rate, and age in coastal parishes.

**Coastal Parish**	**Cumulative Permitted ATUs** as of 2023	**Mean ATU/Housing Unit** 1990 - 2023	**Mean ATU Age** years as of 2023
St. Tammany	32 570	0.491	14.3
Tangipahoa	30 462	1.03	15.4
Calcasieu	23 966	0.696	15.2
Lafayette	21 206	0.495	13.3
Lafourche	18 631	1.49	19.6
Livingston	17 347	0.591	12.6
Ascension	17 115	0.592	14.9
Terrebonne	13 075	1.04	19.4
St. Martin	10 527	2.02	19.1
Vermilion	9814	1.45	17.6
Acadia	9212	1.86	19.0
Iberia	7962	1.44	18.1
Assumption	5382	2.48	21.4
Jefferson Davis	4529	1.61	16.8
St. James	3623	2.04	20.6
Iberville	2950	1.07	15.5
Cameron	2661	1.30	17.4
Jefferson	2080	0.098	7.1
St. Mary	1527	0.581	16.1
Orleans	1100	0.037	4.2
St. Bernard	899	0.162	8.5
St. Charles	527	0.081	8.7
Plaquemines*	354	0.116	--
St. John the Baptist	260	0.068	8.6
**Coastal Parishes**	**237 809**	**0.669**	**14.92**
**Louisiana**	**412 552**	**0.754**	**15.25**

*Projections for Plaquemines parish made using RUCC1 data in the absence of historic data for the parish.

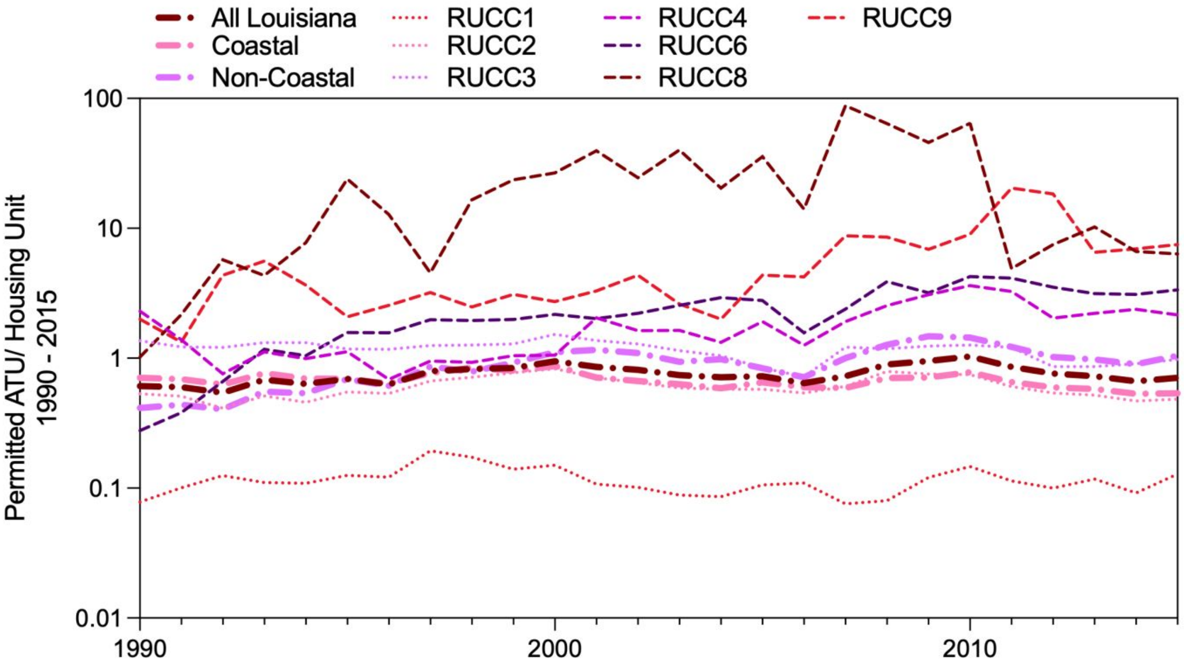

Figure S9. Average permitted ATU/housing unit ratio (*i.e.*, ATU utilization ratio) by stratified by RUCC group, coastal and non-coastal parishes, and all Louisiana parishes.

Table S4. Comparison of annual permitted ATU/housing unit ratios by RUCC, metro vs. non-metro, coastal vs. non-coastal, and all Louisiana parishes.

	**RUCC1**	**RUCC2**	**RUCC3**	**RUCC4**	**RUCC6**	**RUCC8**	**RUCC9**	**LA**	**METRO**	**NON-METRO**	**COASTAL**	**NON-COASTAL**
**RUCC1**	--	>0.9999	**<0.0001**	**<0.0001**	**<0.0001**	**<0.0001**	**<0.0001**	0.0521	0.0996	**<0.0001**	0.3909	**0.0028**
**RUCC2**		--	**0.0277**	**0.0004**	**<0.0001**	**<0.0001**	**<0.0001**	>0.9999	>0.9999	**<0.0001**	>0.9999	>0.9999
**RUCC3**			--	>0.9999	>0.9999	**<0.0001**	**0.0118**	>0.9999	**0.0118**	**0.0229**	0.2482	>0.9999
**RUCC4**				--	>0.9999	**0.0072**	0.4231	0.0608	**<0.0001**	>0.9999	**0.0059**	0.6552
**RUCC6**					--	0.0812	>0.9999	**0.0051**	**<0.0001**	>0.9999	**0.0003**	0.0859
**RUCC8**						--	>0.9999	**<0.0001**	**<0.0001**	0.6027	**<0.0001**	**<0.0001**
**RUCC9**							--	**<0.0001**	**<0.0001**	>0.9999	**<0.0001**	**<0.0001**
**LA**								--	>0.9999	**<0.0001**	>0.9999	>0.9999
**METRO**									--	**<0.0001**	>0.9999	>0.9999
**NON-METRO**										--	**<0.0001**	**<0.0001**
**COASTAL**											--	>0.9999
**NON-COASTAL**												--

Table S5. Demographic and governance characteristics of St. Tammany and Tangipahoa parishes and the state of Louisiana.

Metric	St Tammany	Tangipahoa	Louisiana
Population	269,331	135,218	4,620,000
Poverty (%)	12.20	19.40	18.90
Median Household Income ($)	79,277	57,256	60,023
Total General Revenue ($ million)	245	109	47,957
